# Targeting cellular source-specific CXCL9 signaling for immunotherapy in oral squamous cell carcinoma

**DOI:** 10.3389/fimmu.2026.1768606

**Published:** 2026-02-12

**Authors:** Miao Qiu, Ling Wang, Honglin Tang, Shan Jiang

**Affiliations:** 1 Department of Pharmacy, Shenzhen Stomatology Hospital (Pingshan) of Southern Medical University, Shenzhen, China; 2 Department of Prosthodontics, Shenzhen Stomatology Hospital (Pingshan) of Southern Medical University, Shenzhen, China; 3 Department of Medical Oncology Sir Run Run Shaw Hospital School of Medicine, Zhejiang University, Hangzhou, China; 4 Department of Periodontology, Shenzhen Stomatology Hospital (Pingshan) of Southern Medical University, Shenzhen, China; 5 Shenzhen Clinical College of Stomatology, School of Stomatology, Southern Medical University, Shenzhen, China

**Keywords:** CXCL9, fibroblast, immune checkpoint blockade, oral squamous cell carcinoma, tumor microenvironment

## Abstract

Oral squamous cell carcinoma (OSCC) remains a clinical challenge due to its high recurrence, metastatic potential, and limited responsiveness to current immunotherapies. Within the tumor microenvironment (TME), the C-X-C motif chemokine ligand 9 (CXCL9) plays a pivotal yet paradoxical role, functioning as both an anti-tumor effector and a tumor-promoting factor depending on its cellular origin. This review proposes that the function of CXCL9 is not intrinsic but dictated by the interplay among its cellular source, microenvironmental context, and receptor-expressing cells. We delineate how this tripartite crosstalk influences immune checkpoint blockade (ICB) outcomes through mechanisms such as T-cell suppression, regulatory T cells recruitment, and PD-L1 upregulation. Myeloid cell-derived CXCL9 generally mediates anti-tumor immunity by recruiting cytotoxic lymphocytes, whereas CXCL9 produced by stromal cells like cancer-associated fibroblasts often contributes to metastasis and immune evasion. Given this complexity and unique immunosuppressive and fibrotic properties of OSCC, we argue that simply augmenting or blocking CXCL9 is insufficient. Instead, overcoming ICB resistance in OSCC requires a precision strategy focused on targeting cell-specific CXCL9 signaling. Ultimately, dissecting and therapeutically navigating the source-specific CXCL9 network is essential to transform the OSCC TME and improve clinical outcomes.

## 1 Introduction

Oral squamous cell carcinoma (OSCC) accounts for over 90% of oral malignancies and remains one of the most aggressive head and neck cancers, characterized by frequent local recurrence, lymph node metastasis, and a five-year survival rate below 50% (1). Most OSCC cases are diagnosed at advanced stages with a high risk of metastasis, which is the primary cause of mortality (2, 3). Although surgical resection combined with radiotherapy or chemotherapy remains the standard of care, the survival rate has improved only marginally in recent decades (4). Therefore, elucidating the molecular mechanisms underlying OSCC progression and identifying effective biomarkers for early diagnosis and predicting immunotherapy responsiveness are critical important.

OSCC development is driven by a complex interplay of genetic, epigenetic, and environmental factors, including epithelial-mesenchymal transition (5, 6), angiogenesis or lymphangiogenesis (7–9), oral microbial community (10, 11), long non-coding RNA (12, 13) and the tumor microenvironment (TME) (14). Among these, TME has emerged as a central determinant of tumor progression and therapeutic resistance. The TME comprises non-malignant stromal and immune cells such as macrophages, fibroblasts, neutrophils, dendritic cells, and myeloid-derived suppressor cells (MDSCs), as well as soluble factors like cytokines, chemokines, and extracellular vesicles. Despite growing interest, most TME-targeted strategies remain in preclinical stages, likely due to an incomplete understanding of the dynamic intercellular signaling that shapes immune responses (15).

Chemokines are a subset of chemoattractant cytokines, defined by their ability to stimulate cellular migration and positioning along a chemical gradient within the TME. Their interactions with specific G-protein-coupled receptors orchestrate the recruitment of diverse immune cells, critically shaping anti-tumor immunity while also being co-opted by tumors to promote angiogenesis, metastasis, and an immunosuppressive niche (16, 17). Among them, the CXC subfamily, particularly those lacking ELR motif (ELR^-^), are pivotal in immune cell trafficking. One such pivotal ELR^-^ CXC chemokine is C-X-C motif chemokine ligand 9 (CXCL9) induced by interferon-gamma (IFN-γ). CXCL9 binds with the receptor CXCR3 and recruits CXCR3^+^ cells such as cytotoxic T lymphocytes (CTLs), natural killer cell (NK) cells, and regulatory T cells (Tregs). Through this pathway, CXCL9 can promote both immune activation and suppression, influencing tumor growth, angiogenesis, and metastasis. The transcriptional regulation of CXCL9 involves multiple pathways, including Janus kinase/signal transducer and activator of transcription 1 (JAK/STAT1), nuclear factor κB (NF‐κB), myeloid transcription factor PU.1 (PU.1), multiple myeloma oncogene 1 (MUM1), Fos‐related antigen 1 (Fra-1), and Early growth response‐1(Egr-1) (18). Notably, the receptor CXCR3 exists in three isoforms, CXCR3-A, CXCR3-B, and CXCR3-Alt, which exhibit differences in their expression profiles and biological effects (19–21). While CXCR3-A generally mediates leukocyte migration and immune activation, CXCR3-A on tumor cells can paradoxically enhance tumor progression (22), whereas CXCR3-B exerts anti-proliferative and anti-metastatic effects (23–25). This duality shaped by receptor isoform distribution and the cellular origin of CXCL9 underscores the complex and context-dependent nature of the CXCL9/CXCR3 axis in cancer.

In this review, we highlight the dual roles of CXCL9 within the TME, emphasizing the cell-source–specific determinants of its function. We focus on CXCL9 derived from macrophages, dendritic cells, and fibroblasts-three key cellular compartments that collectively define immune equilibrium in OSCC. We further discuss how these mechanisms contribute to immune checkpoint blockade (ICB) responsiveness and propose that selective modulation of CXCL9 signaling by cellular origin may represent a next-generation approach to improving immunotherapy outcomes in OSCC.

## 2 The dual nature of CXCL9 in the tumor microenvironment

CXCL9 is increasingly recognized as a pivotal but paradoxical mediator within TME, exerting both anti-tumor and pro-tumor functions depending on the cellular and molecular context. This duality arises from its pleiotropic effects on immune cell recruitment, angiogenesis, and tumor cell signaling. Understanding this context-dependent role is crucial for interpreting the divergent outcomes observed across different cancers and therapeutic settings.

### 2.1 CXCL9: an effector or accomplice in disease progression

CXCL9 is widely recognized as a pivotal mediator of anti-tumor immunity and a key determinant of the therapeutic efficacy of ICB, primarily owing to its potent capacity to recruit CXCR3^+^ effector T cells and NK cells (26–28). However, accumulating evidence has revealed a contradictory effect in the function of CXCL9, indicating that it cannot only drive anti-tumor immunity but also mediate immunosuppression and tumor metastasis (29, 30). This apparent contradiction is not an intrinsic property of the molecule itself, but instead reflects the complex interplay among the cellular source of CXCL9, the dynamic TME, and the nature of the receptor-expressing cells. Therefore, a comprehensive understanding of the determinants that shape the functional fate of CXCL9 is essential for harnessing its therapeutic potential. The following sections will dissect this duality by examining the distinct functional outcomes associated with CXCL9 derived from two major cellular compartments: myeloid cells and stromal cells.

### 2.2 Myeloid-derived CXCL9 as an engine of anti-tumor immunity

In immune-activated microenvironment such as those induced by ICB, specific myeloid cell subsets represent the predominant source of protective CXCL9 (26, 31, 32). Within the TME, macrophages are not only more abundant than dendritic cells but also produce substantially higher levels of CXCL9, both in mice and in patients receiving immune checkpoint therapy (33). A distinct pro-inflammatory macrophage population (F4/80^+^MHCII^+^Ly6C^lo^) serves as a critical source of CXCL9, indispensable for the efficacy of anti-PD-L1 therapy through the recruitment of CXCR3^+^ T cells; blockade of CXCL9 abrogates therapeutic response in CT26 tumors (26). It was also reported that CXCL9 increases cytotoxic T lymphocyte (CTL) chemotaxis and inhibits angiogenesis, thereby suppressing tumor growth (34). These M1-hot tumor-associated macrophages (TAMs) facilitated not only the recruitment of CTLs but also the establishment and metabolic maintenance of CD8^+^ tissue-resident memory T cells via CXCL9-mediated signaling, sustaining them through enhanced fatty acid availability, a process linked to prolonged survival (35).

In head and neck squamous cell carcinoma (HNSCC), an immune-hot gene signature composed of CXCL9, CXCL10, CXCL11, and CCL5 predicts responsiveness to ICB (28), underscoring the clinical significance of myeloid-derived CXCL9. Moreover, CD103^+^ conventional type 1 dendritic cells (cDC1s) constitute another non-redundant source (31). Their co-expression of CXCL9 and IL-12 is crucial for NK cell recruitment and activation, which are indispensable for the anti-tumor efficacy of CD47 blockade (36). CXCL9^+^ antigen-presenting cells (CD11c^+^) located within the T-cell zones of tertiary lymphoid structures further shape an immune-activated microenvironment highly responsive to ICB (37). Collectively, myeloid-derived CXCL9 acts as a coordinator of anti-tumor immunity by orchestrating cytotoxic lymphocyte recruitment and activation, ultimately constructing an immune-activated niche that enhances therapeutic efficacy (38).

### 2.3 The tumor-promoting effect of interstitial cells and CXCL9

When CXCL9 is produced by stromal cells or within an immunosuppressive TME, its function can be subverted towards promoting tumor progression. In breast cancer and melanoma models, cancer-associated fibroblast (CAF)-derived CXCL9/10 activates CXCR3 receptors on tumor cells, markedly enhancing their migratory potential and facilitating lung colonization through the JNK-IL-1 signaling axis, constituting a non–immune-dependent mechanism of metastasis (30, 39, 40). Similarly, CXCL9 expressed by tumor vascular endothelial cells exhibits potent chemotactic activity toward melanoma cells, thereby promoting distant metastasis (41). These represent a stark deviation from its canonical immune recruiting role.

Beyond its direct tumor-promoting effects, CXCL9 can also be hijacked to foster immune suppression. For instance, CXCL9 secreted by BATF3^+^ dendritic cells recruits CXCR3^+^ regulatory T cells into the TME, dampening antitumor immunity and accelerating tumor progression (29). In bladder cancer, tumor-associated dendritic cells produce high levels of CXCL9 that upregulate PD-L1 expression on T24 tumor cells through CXCR3-mediated signaling, thus promoting tumor growth (42). Moreover, the immunosuppressive milieu itself can extinguish protective CXCL9 signaling. Transforming growth factor β1 (TGF-β1), a key driver of immune evasion and therapeutic resistance, epigenetically silences CXCL9/10 expression in fibroblasts through chromatin remodelings (43). Altogether, CXCL9 may contribute to tumor progression either by recruiting suppressive Tregs or invasive cancer cells, or by being silenced within an immunosuppressive microenvironment.

### 2.4 The crosstalk between source, microenvironment and receiver determines the function of CXCL9

**Figure 1:**
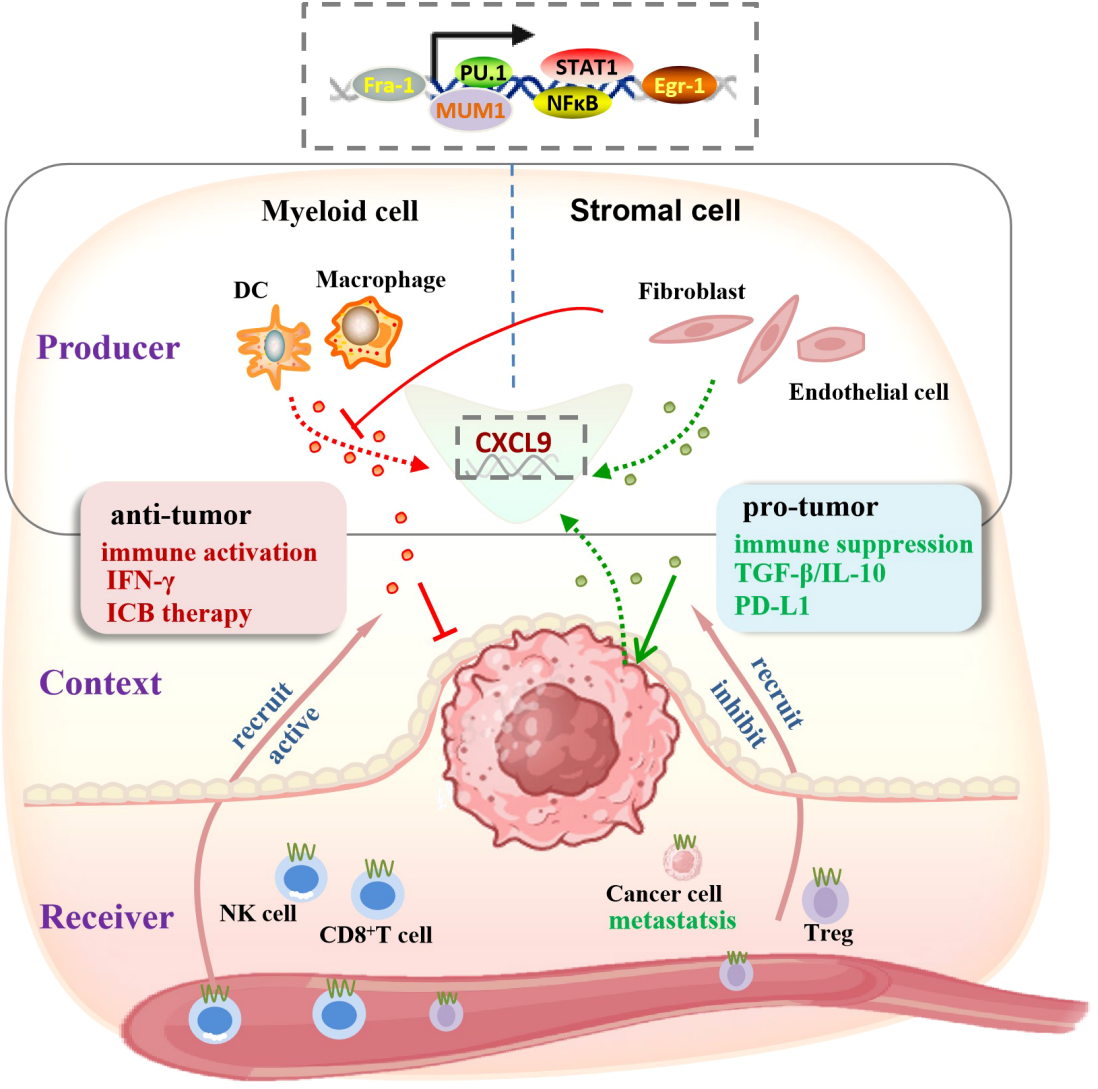
The crosstalk between source, microenvironment and receiver determines the function of CXCL9. CXCL9 function is determined by the convergence of three elements: (1) Producer (the cellular source): myeloid cells (dendritic cells, macrophages) secrete CXCL9 to mediate anti-tumor responses, while stromal cells (fibroblasts, endothelial cells) produce CXCL9 that contributes to pro-tumor processes; (2) Context (the tumor microenvironment): the immune-activating context (IFN-γ, ICB therapy) enhances CXCL9 secretion by myeloid cells; conversely, the immune-suppressive context (e.g., TGF-β) inhibits CXCL9 production in stromal cells, reshaping the balance of CXCL9-mediated effects; (3) Receiver (the target cell): engagement with CTLs or NK cells drives tumor elimination, however, ligation to CXCR3 on Tregs and cancer cells exerts pro-tumor/immune suppression effect. This model explains the opposing roles of CXCL9 in tumor progression and immunity. The top dashed box indicates the transcription factors governing CXCL9 expression.

The biological outcome of CXCL9 signaling is ultimately shaped by the interplay among its cellular source, the surrounding microenvironment, and the receptor-bearing cells (**Figure 1**). Most studies demonstrate that CXCL9 enhances anti-tumor immunity and ICB responsiveness primarily by recruiting CD8^+^T cells. Myeloid-derived CXCL9, particularly from macrophages or dendritic cells, is generally associated with protective immunity (28, 35), whereas stromal cell-derived CXCL9, originating from CAFs or endothelial cells, is frequently linked to tumor progression and metastasis (30, 40, 41).

Nevertheless, this dichotomy is not absolute, as cellular states are highly plastic and environmentally regulated. Certain dendritic cell subsets can switch to an immunosuppressive phenotype, producing CXCL9 that recruits Tregs or induces PD-L1 expression (29, 42). This highlights the decisive role of the TME, which orchestrates CXCL9 expression and function. Immunostimulatory signals such as IFN-γ or ICB treatment promote CXCL9 production in immune cells (26, 35), whereas immunosuppressive cytokines, notably TGF-β, suppress or reprogram CXCL9 activity through epigenetic silencing, particularly within stromal compartments (43, 44).

Ultimately, the biological consequence depends on the CXCR3^+^ recipient cell. Engagement with CTLs or NK cells drives tumor elimination, however, ligation to CXCR3 on Tregs suppresses effector immune responses (29), and if engagement with tumor cells expressing CXCR3 directly promotes invasion and metastatic dissemination (22, 30, 39). Therefore, the multifaceted effects of CXCL9 are defined by a triad of determinants-its source, the microenvironmental context, and the receptor cell population.

## 3 What is the effect of CXCL9 in the complex microenvironment of OSCC?

The OSCC tumor microenvironment exhibits distinctive anatomical and biological features, shaped by three interrelated determinants (10, 45, 46). First, it is characterized by intense fibrosis and stromal desmoplasia, which leads to a dominant presence of CAFs. Second, it engages in a persistent crosstalk with a diverse and spatially constrained oral microbiome that exerts direct regulatory effects on local immune responses. Third, OSCC arises within a specialized mucosal immune niche that is structurally and functionally distinct from those of other head and neck cancer sites. Collectively, these contextual features critically shape the spatiotemporal activity and functional interpretation of chemokine signaling pathways, including CXCL9.

Emerging evidence, particularly from HPV-negative HNSCC, reinforces the classic paradigm of CXCL9 as a cornerstone of immunologically “hot” tumors. Spatial multiomics studies confirm that CXCL9 upregulation is a core feature of highly immune-active tumors, where it correlates with active interferon signaling, enhanced antigen presentation, and crucially, the spatial co-localization of CD8^+^ T cells with tumor cells (47). In this context, CXCL9 production by DCs and TAMs is understood to promote CD8^+^ T cell recruitment, activation, and tumor cell killing, fostering a favorable antitumor microenvironment (28, 48).

However, the functional role of the chemokine CXCL9 transcends its classical function in recruiting Th1 and T cells and is instead reprogrammed by TME to serve as a critical mediator of immune evasion in OSCC. This functional reprogramming is primarily mediated by a distinct subpopulation of CAFs that express CXCL9, thereby establishing a direct link between the characteristic interstitial fibrosis observed in OSCC and the suppression of antitumor immunity. Single-cell RNA sequencing studies have identified a TDO2^+^ myofibroblast subset within OSCC that secretes chemokines such as CXCL9 to recruit T cells to peritumoral regions. However, it induces the differentiation of CD4^+^ T cells into Tregs and drives CD8^+^ T-cell dysfunction, thereby hijacking CXCL9-mediated recruitment to establish localized immunosuppressive niches (49). More directly, spatial transcriptomic analyses in HNSCC (which includes OSCC) have revealed MHC-I^hi^Galectin-9^+^ (Gal9^+)^ CAFs. This subset highly expresses CXCL9, CXCL10, and CXCL12. It uses CXCL9 to recruit CD8^+^ T cells into its vicinity, then employs its surface-bound Gal9 to directly induce T-cell exhaustion. This action spatially forms a “trap” that restricts productive T-cell infiltration into tumor islets (50). Furthermore, tumor cell-derived fibroblast growth factor-2 (FGF-2) is identified as an upstream regulator of CXCL9 in OSCC. FGF-2 promotes lymphangiogenesis and, critically, stimulates the secretion of CXCL9. This cascade mediates the recruitment and subsequent egress of CD8^+^ T cells via newly formed lymphatic vessels, directly contributing to an immune-cold TME and poorer patient prognosis (51).

Notably, the regulation of CXCL9 expression by the oral microbiome also appears to exhibit this context-dependent, bidirectional nature. Specifically, periodontal pathogens represented by *Porphyromonas gingivalis* have been demonstrated to suppress Th1-type chemokines including CXCL9 and contribute to an immunosuppressive tumor microenvironment in OSCC (52). Conversely, evidence from other tumor types indicates that selected commensal bacteria or probiotics can enhance anti-tumor immunity by activating myeloid cells and promoting CXCL9 production, suggesting that microbial effects on the CXCL9 axis are highly context-dependent (53–55). Consequently, the integrated impact and dominant regulatory function of the entire oral microbial community on the CXCL9 network in OSCC is an area of active investigation.

**Figure 2:**
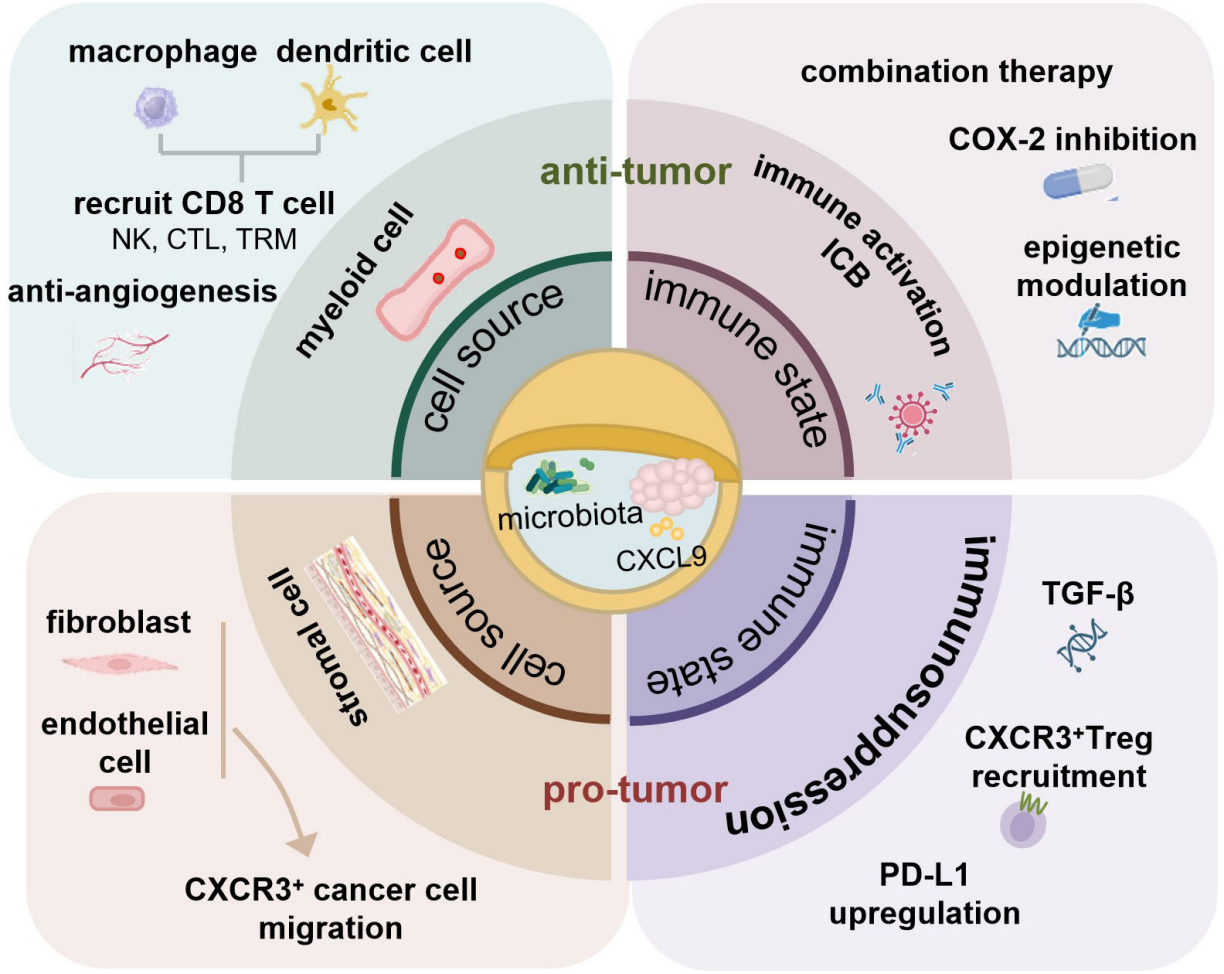
The functional fate of CXCL9 in the OSCC tumor microenvironment. The OSCC microenvironment is characterized by profound immunosuppression, intense fibrosis, and constant exposure to a unique oral microbiome. CXCL9 signaling in OSCC is determined by the integration of three elements, often skewed toward a pro-tumor outcome. Stromal cells (e.g., fibroblasts) produce CXCL9 that directly activates CXCR3 on tumor cells, driving invasion and metastasis. In contrast, myeloid cells (e.g., macrophages, DCs) produce CXCL9 that recruits and activates cytotoxic CD8^+^ T cells, initiating anti-tumor immunity. A dominant immunosuppressive environment favors the pro-tumor axis. Successful intervention, such as immune checkpoint blockade, can shift the balance to an immune-activated environment, unleashing the anti-tumor potential of CXCL9 and resulting in immune-mediated tumor killing. The net effect of the complex oral microbiome on the CXCL9 axis is context-dependent and an area of active investigation. The model highlights that therapeutic success depends on suppressing the pro-tumor axis (red) while promoting the anti-tumor axis (green).

This dichotomy presents a critical question for OSCC: whether the CAFs and immunosuppressive microbiota completely dominate CXCL9-mediated tumor progression, or whether protective signals derived from myeloid cells could be unmasked? To visualize and address this central question, we have developed a model presented in **Figure 2**, which illustrates how the functional fate of CXCL9 is governed by the dynamic interplay of opposing forces. Understanding this balance is essential for developing therapeutic strategies aimed at modulating the CXCL9-CXCR3 axis to enhance anti-tumor immunity while mitigating its pro-tumorigenic effects. Such insights directly motivate the exploration of CXCL9-targeted approaches in overcoming ICB resistance, as discussed in the following section.

## 4 Targeting the CXCL9-CXCR3 axis to overcome immunotherapy resistance in OSCC

ICB, particularly antibodies against PD-1/PD-L1, represents a breakthrough in cancer therapy, supplementing surgery, radiotherapy, and chemotherapy (56). By blocking PD-1/PD-L1 signaling, ICB alleviates immunosuppression on T lymphocytes, leading to enhanced CTL activation and, in some cases, remarkable tumor regression (57, 58). However, only a minority of OSCC patients achieve clinical benefit, with response rates ranging from 6.5% to 21.8% in recurrent or metastatic head and neck squamous cell carcinoma (59). The limited efficacy is largely attributed to the “cold” tumor phenotype, characterized by poor infiltration of effector CD8+ T cells or abundant accumulation of Tregs (60–62). Other contributing factors include impaired antigen presentation, immunosuppressive tumor microenvironment, and microbiome composition (63–66). These obstacles underscore the urgent need to identify biomarkers predictive of ICB responsiveness and develop strategies to enhance anti-tumor immunity in OSCC.

The chemokine CXCL9 plays a pivotal role in shaping the immune landscape of OSCC and influencing ICB outcomes. CXCL9 produced by myeloid cells, such as macrophages and dendritic cells, recruits and activates CD8^+^ T cells and NK cells, thus promoting an “immune-hot” TME conducive to effective checkpoint inhibition (26, 35, 38). Conversely, CXCL9 derived from stromal cells, including CAFs and vascular endothelial cells, may contribute to immunosuppression or tumor progression, potentially dampening the therapeutic benefits of ICB (29, 30, 40, 41). These findings highlight the dual role of CXCL9 in OSCC and suggest that its expression and source within the TME can serve as a biomarker for predicting ICB responsiveness.

A substantial and multi-tiered body of evidence supports the therapeutic modulation of the CXCL9-CXCR3 axis. As summarized in **Table 1**, this evidence spans direct clinical interventions to foundational mechanistic studies, collectively informing a precision oncology framework. Although definitive late-phase clinical trial data specific to OSCC are still emerging, the existing body of evidence is robust and compelling. First, direct interventional strategies aim to remodel the local immune microenvironment through focal delivery or induction of CXCL9. These strategies encompass a range of approaches, such as intratumoral delivery of CXCL9 via oncolytic viruses in HNSCC, engineered nanoparticles that deliver CXCL9-encoding circular RNA with anti-PD-1 single-chain variable fragment (scFv) (67), co-delivery CXCL9 with BRD4-PROTAC(dBET6) (68), the use of recombinant adeno-associated virus (AAV) vectors for focal and sustained CXCL9 expression (69) and the application of CXCL9/10-engineered DCs therapy combined with ICB (70). Second, clinical-translational insights reveal key regulatory nodes. For instance, somatic NCOA3 mutations suppressed CXCL9 expression and CD8^+^ T-cell infiltration via the HSP90α/EZH2 axis, highlighting tumor-intrinsic control of this chemokine network (71). Third, preclinical studies solidify stromal-derived CXCL9 as an actionable target. In melanoma and OSCC models, inhibition of CXCL9/10 secretion from fibroblasts was shown to attenuate metastasis (40, 49). Moreover, THBS2-expressing matrix CAFs suppress myeloid-derived CXCL9/10 to limit CXCR3^+^CD8^+^T-cell recruitment, an effect that can be reversed by blocking this axis (72). Collectively, these diverse approaches share the unified objective of rebalancing the CXCL9 network to overcome ICB monotherapy resistance.

**Table 1:** Evidence landscape for targeting the CXCL9/CXCR3 axis.

Intervention	Mechanisms related to CXCL9/CXCR3	Cancer	Research type	Implication
CXCL9/CXCL10-expressing oncolytic virus + anti-PD1	Local delivery of CXCL9/10 to enhance T-cell recruitment	metastatic epithelial tumors, HNSCC	early-phase clinical Trial (Phase I) (NCT05043714, NCT04830592)	Direct supplement beneficial CXCL9
CXCL9/10- engineered dendritic cells + ICB	Adoptive cell therapy locally provides CXCL9 signaling	HNSCC (70)	Preclinical	Cell therapy pathway of supplementing beneficial signals
Nanoparticles deliver CXCl9-encoded circRNA & scFv	Nanocarriers enable targeted co-delivery of CXCL9 and immune drugs	Breast cancer (67)	Preclinical	Collaborative delivery and targeted delivery
nChap platform collaborative delivery of CXCL9 and BRD4-PROTAC (dBET6)	Chemical factor induction works in synergy with epigenetic reprogramming	Breast cancer (68)	Preclinical	Synergistic treatment of immune recruitment plus suppression relief
Recombinant AAV-mediated CXCL9 gene therapy	Achieve long-term and stable expression of CXCL9 locally in tumors	Glioblastoma (69)	Preclinical	Verify the feasibility of local sustained expression
Engineered Salmonella delivery CXCL9/CCL2	CXCL9 and CCL2 are expressed *in situ* and continuously within the tumor	Osteosarcoma (73)	Preclinical	Live bacterial vectors enable the continuous production of CXCL9 locally in tumors
TLR9 agonist + anti-PD-1	Upstream induction of CXCL9/10 via IFN-α from pDCs.	melanoma, HNSCC	Clinical Trial (Phase III, NCT03445533; Phase II, NCT02521870)	Indirect induce endogenous CXCL9
NCOA3 mutation	Wild-type inhibits, while mutant type upregulates the expression of CXCL9	colon cancer (71)	Clinical and TCGA data	Target tumor-intrinsic suppression to activate CXCL9
Epigenetic modulation (EZH2/DNMT1 inhibition)	Reversal of epigenetic silencing of CXCL9/10	ovarian cancer (74)	Preclinical/Translational	Derepress CXCL9 transcription
Combination ICB (e.g., αTIM-3 + αPD-1)	Enhances cDC1-derived CXCL9/10 via IFN-γ	breast cancer (75)	Preclinical	Synergize DC-derived CXCL9
Inhibition of CAF-derived CXCL9 signaling	CXCL9 from CAFs activates CXCR3 on tumor cells to drive migration and metastasis	melanoma (40) OSCC (49)	Preclinical	Block pro-metastatic CAF signaling

HNSCC, Head and Neck Squamous Cell Carcinoma; ICB, Immune Checkpoint Blockade; scFv, single-chain variable fragment; nChap, nanochaperone, BRD4-PROTAC (dBET6), Protein Degradation Targeting chimera for BRD4, compound dBET6; AAV, Adeno-Associated Virus; TLR9, Toll-like receptor 9; TCGA, The Cancer Genome Atlas; NCOA3, Nuclear receptor coactivator 3; EZH2, Enhancer of zeste homolog 2; DNMT1, DNA methyltransferase 1; αTIM-3, T-cell immunoglobulin and mucin domain containing (TIM)-3 blocking antibodies; IFN-γ, interferon-gamma; CAF, cancer-associated fibroblast; OSCC, oral squamous cell carcinoma.

The heterogeneous responses to immunotherapy in OSCC can be fundamentally traced to interpatient variability in the dominant cellular source of CXCL9. To address this, we propose a “cellular source-determined” precision framework, which is particularly compelling in OSCC given its unique pathological context: the prevalent fibrotic stroma establishes stromal cells as a critical therapeutic target, while the anatomical accessibility of the oral cavity favors localized, spatially-informed interventions. This framework is implemented through spatial profiling techniques. Spatial transcriptomics (10x Visium) and multiplex immunofluorescence (mIF) can be employed to directly visualize and quantify CXCL9 expression in relation to specific cellular markers, such as α-smooth muscle actin (α-SMA), fibroblast activation protein (FAP), and Gal9 for fibroblasts, or CD68 and CD11c for myeloid cells (47, 50, 72). These approaches allow precise discrimination of CXCL9 cellular origin within intact tumor architecture, thereby clarifying its context-dependent function.

This spatial profiling enables molecular stratification, which provides a rational basis for therapeutic decision-making. A “myeloid-dominant” profile supports strategies aimed at reinforcing immune activation, whereas a “stroma-dominant” profile suggests that modulation of the stromal compartment should precede or accompany immune checkpoint blockade. This closed-loop, biomarker-guided strategy represents a precision medicine approach that is particularly suited to overcoming immune exclusion and ICB resistance in OSCC.

In conclusion, overcoming immunotherapy resistance in OSCC requires a nuanced understanding of the CXCL9-CXCR3 axis. By adopting a precision medicine framework that integrates spatial profiling with source-specific therapeutic design, it becomes possible to transform a traditionally immune-resistant tumor into one that is amenable to immunomodulation.

## 5 Conclusion

The function of the chemokine CXCL9 in cancer is not predetermined but is principally determined by its cellular source within the tumor microenvironment. This review establishes that distinguishing between CXCL9 derived from anti-tumor myeloid cells versus pro-tumor stromal cells is critical for understanding its duality. Specifically, CXCL9 derived from myeloid lineages, such as macrophages and dendritic cells, generally serves as a cornerstone of anti-tumor immunity by recruiting and activating effector lymphocytes. In contrast, CXCL9 produced by stromal cells, including CAFs and endothelial cells, often participates in pro-tumorigenic processes, notably metastasis. In is characterized by an immunosuppressive and highly fibrotic TME, metastasis-promoting signals originating from the stromal cells may predominate, potentially undermining the therapeutic benefits of immune activation by myeloid lineages. Importantly, under specific conditions such as during ICB therapy, the immune-activating properties of CXCL9 may demonstrate significant therapeutic potential.

Therefore, research should transition from merely evaluating CXCL9 expression levels to identifying its cellular sources within the OSCC TME. The therapeutic objective is not to indiscriminately inhibit or augment CXCL9, but to develop precision strategies that selectively target its detrimental sources while preserving or enhancing its beneficial functions. This source-specific approach holds significant promise for the rational design of CXCL9-targeted therapies and combined therapy in OSCC.
